# Organic Tracers from Asphalt in Propolis Produced by Urban Honey Bees, *Apis mellifera* Linn.

**DOI:** 10.1371/journal.pone.0128311

**Published:** 2015-06-15

**Authors:** Abdulaziz S. Alqarni, Ahmed I. Rushdi, Ayman A. Owayss, Hael S. Raweh, Aarif H. El-Mubarak, Bernd R. T. Simoneit

**Affiliations:** 1 Department of Plant Protection, College of Food and Agriculture Sciences, King Saud University, Riyadh, 11451, Saudi Arabia; 2 Chair of Green Energy Research, College of Food and Agriculture Sciences, King Saud University, Riyadh, 11451, Saudi Arabia; 3 College of Earth, Oceanic and Atmospheric Sciences, Oregon State University, Corvallis, Oregon, 97333, United States of America; 4 Department of Earth and Environmental Sciences, Sana’a University, Sana’a, Yemen; 5 Department of Chemistry, Oregon State University, Corvallis, Oregon, 97331, United States of America; University of British Columbia, CANADA

## Abstract

Propolis is a gummy material produced by honey bees to protect their hives and currently has drawn the attention of researchers due to its broad clinical use. It has been reported, based only on observations, that honey bees also collect other non-vegetation substances such as paint or asphalt/tar to make propolis. Therefore, propolis samples were collected from bee hives in Riyadh and Al-Bahah, a natural area, Saudi Arabia to determine their compositional characteristics and possible sources of the neutral organic compounds. The samples were extracted with hexane and analyzed by gas chromatography-mass spectrometry. The results showed that the major compounds were n-alkanes, n-alkenes, methyl n-alkanoates, long chain wax esters, triterpenoids and hopanes. The n-alkanes (ranging from C_17_ to C_40_) were significant with relative concentrations varying from 23.8 to 56.8% (mean = 44.9+9.4%) of the total extracts. Their odd carbon preference index (CPI) ranged from 3.6 to 7.7, with a maximum concentration at heptacosane indicating inputs from higher plant vegetation wax. The relative concentrations of the n-alkenes varied from 23.8 to 41.19% (mean = 35.6+5.1%), with CPI = 12.4-31.4, range from C_25_ to C_35_ and maximum at tritriacontane. Methyl n-alkanoates, ranged from C_12_ to C_26_ as acids, with concentrations from 3.11 to 33.2% (mean = 9.6+9.5%). Long chain wax esters and triterpenoids were minor. The main triterpenoids were α- and β-amyrins, amyrones and amyryl acetates. The presence of hopanes in some total extracts (up to 12.5%) indicated that the bees also collected petroleum derivatives from vicinal asphalt and used that as an additional ingredient to make propolis. Therefore, caution should be taken when considering the chemical compositions of propolis as potential sources of natural products for biological and pharmacological applications. Moreover, beekeepers should be aware of the proper source of propolis in the flight range of their bee colonies.

## Introduction

Honey bees collect waxy/resinous/gummy substances from different parts of plants, such as buds, leaves, stems and flowers, to make a sticky material known as propolis [1], which they utilize to protect their hives from invaders and infection by bacteria and fungi [2]. Furthermore, honey bees use the propolis to regulate nest temperature, light and humidity for optimum conditions [3,4]. In ancient times, propolis was used as a remedy against some diseases [5]. Recent studies have shown that propolis has biological activities, which are related to its different chemical compositions [6]. Thus, propolis has drawn the attention of researchers due to its broad clinical use as an antibiotic against emerging strains of pathogens resistant to synthetic antibiotics. The assorted chemical compositions and biological activities of propolis depend on the plant sources and collecting season [5]. The three possible sources of the organic components of propolis are plants, secreted substances from honey bee metabolism, and extraneous materials introduced during propolis formulation [1]. Propolis is normally composed of resin and vegetable balsam (50%), wax (30%), essential and aromatic oils (10%), pollen (5%) and other substances (5%) [7,8]. Therefore, the variations in chemical composition and biological activities of propolis gathered from different geographical locales are important for pharmaceutical purposes [9].

Some researchers and apiarists reported that honey bees would likely collect other substances such as paints, mineral oils or asphaltic tar to make bee glue-like substance (propolis) when vegetation was scarce in an area [10–12]. The researchers of this work have observed honey bees collecting matter from asphalt, which was also observed by others [12]. Saudi Arabia is located in western Asia with arid climatic conditions that endowed the country with a limited number of flowering plants. Few studies have been conducted to investigate the chemical components of propolis from Saudi Arabia [13].

The purposes of this study are to determine the characteristics and sources of the organic chemical components of propolis made by honey bees in an arid urban region, and to examine if the honey bees are specifically selective in collecting ingredients to make propolis or collect any sticky substance from diverse sources including anthropogenic materials. Thus, we collected propolis from bee hives in the city of Riyadh, Saudi Arabia, where construction activities including road paving (presence of fresh asphalt) are in progress and the locale lacks resinous plants.

## Methodology

### Propolis and asphalt sampling

**Fig 1:**
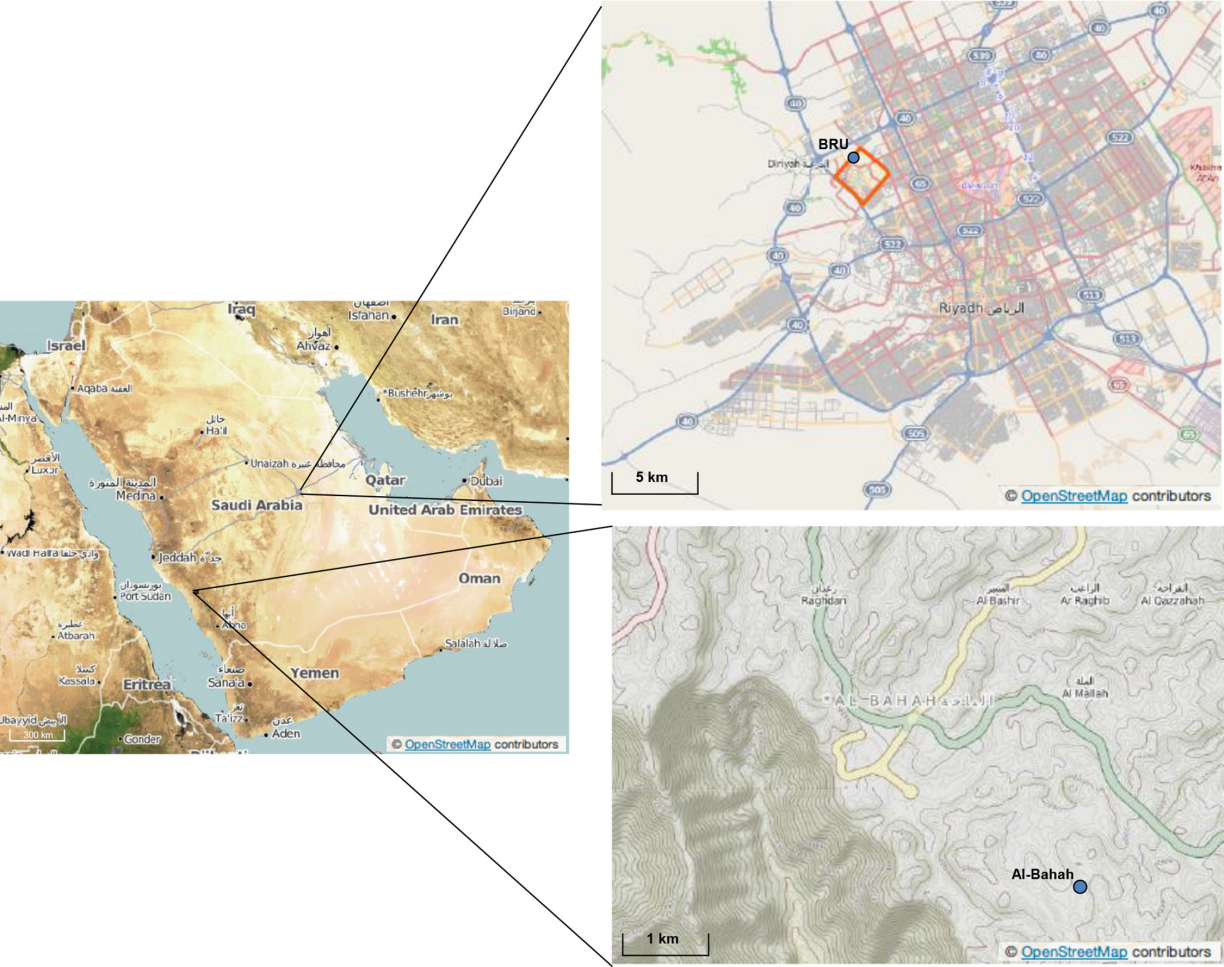
Map showing the location of the propolis sample collection in Riyadh (BRU) and Al-Bahah, Saudi Arabia (“OpenStreetMap contributors”, http://www.openstreetmap.org).

Propolis samples were collected from honey bee colonies at the Bee Research Unit (BRU), King Saud University in Riyadh. The site is located in the northeastern outskirts of the city (Fig 1) and observed to have less vegetation cover with heavy construction activities including road paving with asphalt. A control propolis sample was collected from the rural region of Al-Bahah in southwestern Saudi Arabia (Fig 1). Propolis was sampled from replicated honey bee colonies of the indigenous race, *Apis mellifera jemenitica*, reared in modern Langstroth hives. The samples were collected from propolis adhering above and between combs by scratching with a pre-cleaned stainless steel spatula. Any hive impurities were excluded and samples were stored in glass jars and transferred to the laboratory for chemical analysis. Also, a fresh asphalt sample was collected from the surroundings of the apiary where construction activities were in progress.

### Soil and atmospheric particulate matter (PM) sampling

Surface oil and atmospheric particulate matter (PM) samples were collected from different sites in the city of Riyadh to characterize their solvent extractable lipid contents by gas chromatography-mass spectrometry (GC-MS) analysis. The first surface soil (D-S) sample was collected from a low traffic area in King Saud University near the honey bee hives at the BUR, and the second (O-S) sample was from a heavy traffic area in the city center (S1 Fig). The atmospheric PM (D-PM) sample was taken on the roof of a building (~10 m above ground) near the BUR (S1 Fig). The surface soil samples were taken by scraping the uppermost layer (~1 cm) of soil in a 30 cm^2^ area of exposed surface, whereas the PM sample was collected on a quartz fiber filter (QMA, 20.3 x 25.4 cm) using a high volume air sampler. Before sampling, filters were heated to 600°C in order to lower their background contaminant levels, and then placed in cleaned pre-extracted aluminum containers. A weighed filter was put in the sampler and the PM was collected at flow rate of 1.2 m^3^ min^-1^ for about 24 hours.

### Sample extraction

About 5 g of each propolis sample was extracted three times ultrasonically with 20 mL of hexane for a 15 min period each in a 150 mL precleaned and annealed beaker. Hexane dissolves mainly non-polar compounds such as hydrocarbons. The combined extract was filtered through an annealed glass fiber filter to remove the undissolved propolis particles. The filtrate was first concentrated on a rotary evaporator and then reduced using a stream of dry nitrogen gas to a volume of approximately 200 μL. The volume was finally adjusted to exactly 500 μL by hexane. About 5 g of fresh asphalt sample was rinsed with about 15 ml hexane in a precleaned and annealed beaker, and filtered using a glass fiber filter. The filtrate was also adjusted to a 500 μL final volume.

After air drying, each soil sample was sieved to obtain the fine particles (<125 μm) before extraction of the total soluble organic matter (SOM). The extraction for soil and filter was performed twice by adding a mixture of dichloromethane/methanol (40 mL 3:1 v/v) to about 5 g of the particles of each soil sample and the filter, ultra-sonicating for 20 min, and then filtering through pre-extracted glass microfiber filters (*Whatman*, GF/A filters). Each total SOM extract was concentrated under nitrogen blow-down at room temperature to approximately 1.0–1.5 mL before GC-MS analysis.

### Instrumental Analysis

The chemical analysis was carried out by GC–MS with a Hewlett-Packard 6890 gas chromatograph coupled to a 5973 Mass Selective Detector, using a DB-5MS (Agilent) fused silica capillary column (30 m x 0.25 mm i.d., 0.25 μm film thickness) and helium as carrier gas. The GC was temperature programmed from 65°C (2 min initial time) to 310°C at 6°C min (isothermal for 20 min final time) and the MS was operated in the electron impact mode at 70 eV ion source energy. Full scan mass spectrometric data were acquired and processed using the GC–MS ChemStation data system.

### Identification and Quantification

The compounds were identified using the GC-MS response data (i.e. key ion fragmentograms and mass spectra). Retention times and response factors were compared with those of external authentic standards including n-alkanes, n-alkenes, methyl n-alkanoates, sterols, terpenoids, and literature and library data. Unknown compounds were characterized by interpretation of the fragmentation pattern of their mass spectra. The identification of n-alkanes, n-alkenes, n-alkanals, methyl n-alkanoates, wax esters, triterpenoids and hopane biomarkers were based mainly on their mass spectra [i.e. key ions *m/z* 85, 97, 82, 74/87, 257, 218/189 and 191, respectively], and gas chromatographic retention times. Quantifications (relative concentrations of compounds in %) were performed on the peak areas of the compounds derived from the GC-MS total ion or key ion chromatogram profiles. Average response factors were calculated for each compound.

### Statistical analysis

The data set was statistically analyzed by cluster analysis and principal component analysis (PCA) techniques using the SPSS (IBM-Statistical Package for the Social Sciences, version 21) software to assess the similarity and dissimilarity among the different propolis samples.

## Results and Discussion

**Fig 2:**
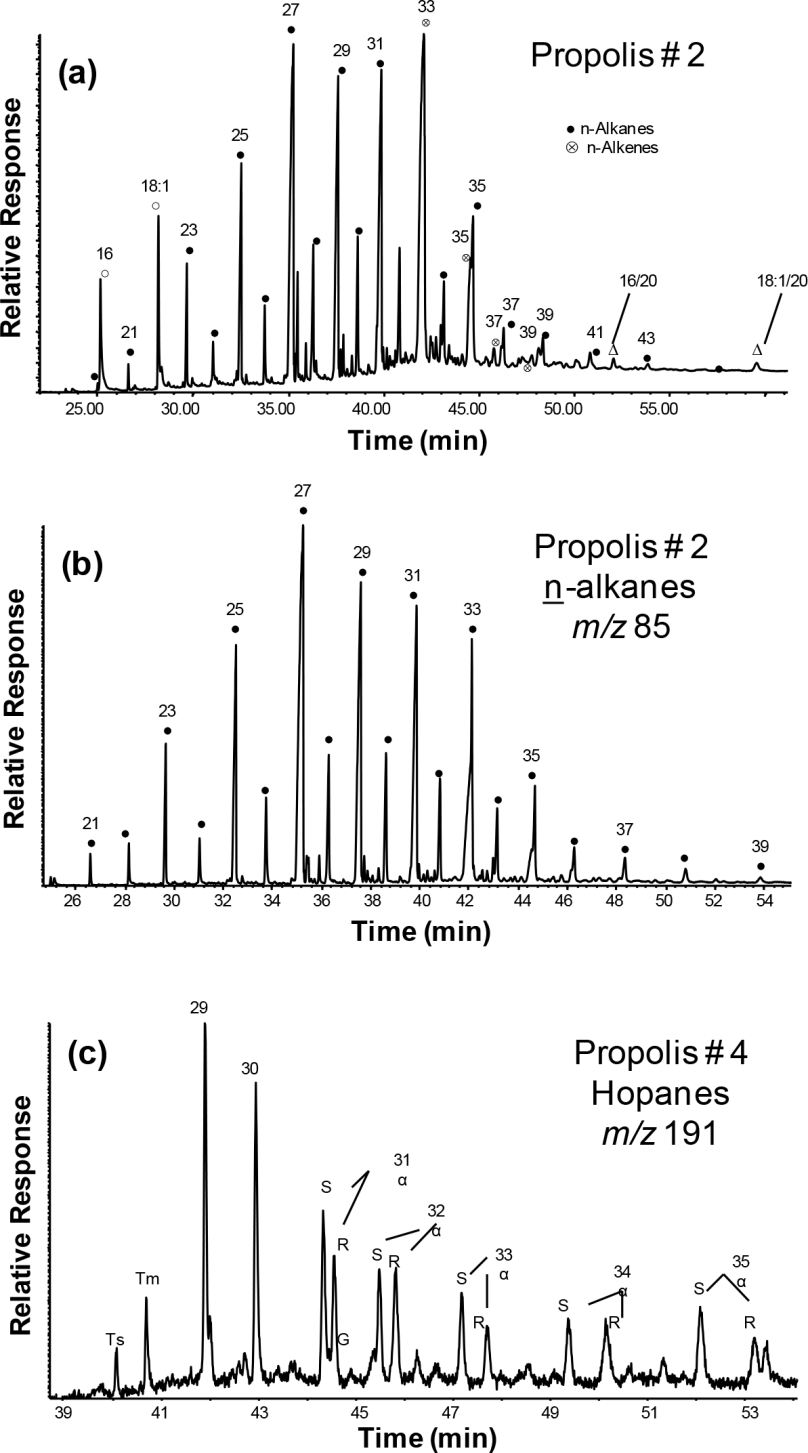
GC-MS total ion current (TIC) trace showing the major organic tracers in propolis sample D2 (a) and typical GC-MS key ion plots for (b) n-alkanes and (c) hopanes.

**Fig 3:**
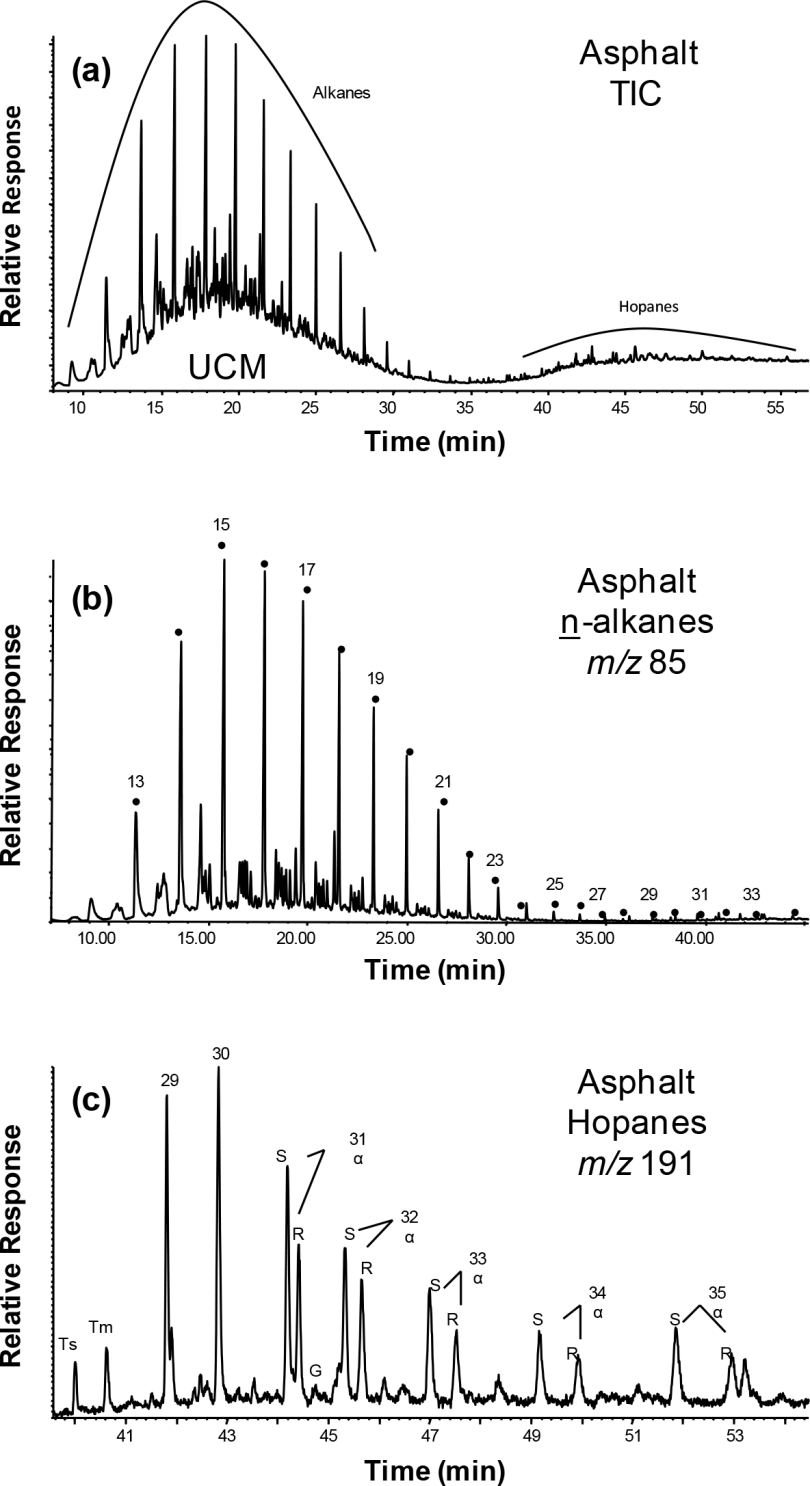
TIC trace showing the major organic tracers in the asphalt sample (a) and typical key ion plots for (b) n-alkanes and (c) hopanes.

The general features of the GC-MS results for the total hexane extractable organic matter (EOM) from the propolis and asphalt samples are shown in Figs 2 and 3, respectively. The major compounds identified were n-alkanes, n-alkenes, n-alkanals, methyl n-alkanoates, wax esters, triterpenoids and hopanes, and their relative concentrations are given in Table 1. The presence and distribution patterns of these compounds in propolis can be utilized to identify their sources. Accordingly, comparisons are possible between known sources and observed organic compound mixtures in the propolis and asphalt samples.

**Table 1:** Relative concentrations (%) of the different compounds from various propolis and asphalt samples collected from Riyadh (D) and Al-Bahah (C), Saudi Arabia.

Compound	Composition	M.W.	D1	D2	D3	D4	D5	D6	D7	D9	D10	D11	C	Mean	SD	Asphalt
**n** **-Alkanes**																
Dodecane	C_12_H_26_	170	N.D.	N.D.	N.D.	N.D.	N.D.	N.D.	N.D.	N.D.	N.D.	N.D.	N.D.			1.1
Tridecane	C_13_H_28_	184	N.D.	N.D.	N.D.	N.D.	N.D.	N.D.	N.D.	N.D.	N.D.	N.D.	N.D.			3.77
Tetradecane	C_14_H_30_	198	N.D.	N.D.	N.D.	N.D.	N.D.	N.D.	N.D.	N.D.	N.D.	N.D.	N.D.			6.18
Pentadecane	C_15_H_32_	212	N.D.	N.D.	N.D.	N.D.	N.D.	N.D.	N.D.	N.D.	N.D.	N.D.	N.D.			7.23
Hexadecane	C_16_H_34_	226	N.D.	N.D.	N.D.	N.D.	N.D.	N.D.	N.D.	N.D.	N.D.	N.D.	N.D.			6.48
Heptadecane	C_17_H_36_	240	0.02	N.D.	N.D.	N.D.	N.D.	N.D.	N.D.	N.D.	N.D.	N.D.	N.D.			5.09
Octadecane	C_18_H_38_	256	0.06	N.D.	N.D.	N.D.	N.D.	N.D.	N.D.	0.06	0.01	N.D.	N.D.			3.58
Nonadecane	C_19_H_40_	268	0.11	N.D.	N.D.	0.12	N.D.	0.09	0.05	0.1	0.06	N.D.	N.D.			2.56
Eicosane	C_20_H_42_	282	0.15	0.13	0.12	0.24	N.D.	0.09	0.09	0.13	0.06	N.D.	0.63			1.83
Heneicosane	C_21_H_44_	296	0.27	0.25	0.24	0.24	0.24	0.09	0.27	0.26	0.34	0.26	0.63			1.25
Docosane	C_22_H_46_	310	0.55	0.38	0.48	0.83	0.24	1.06	0.51	1.74	0.33	0.53	3.04			0.79
Tricosane	C_23_H_48_	324	0.83	1.76	0.84	0.83	1.7	0.53	1.09	0.98	1.74	1.46	2.14			0.42
Tetracosane	C_24_H_50_	338	0.31	0.5	0.36	0.48	0.36	0.18	0.74	0.32	0.41	0.26	1.64			0.22
Pentacosane	C_25_H_52_	352	4.58	4.27	2.65	2.61	4.61	2.03	3.69	3.95	4.62	3.71	10.00			0.13
Hexacosane	C_26_H_54_	366	0.54	1.26	0.72	0.83	0.73	0.26	1.62	0.69	1.12	0.79	2.93			0.08
Heptacosane	C_27_H_56_	380	**9.57**	**13.31**	**8.81**	**9.62**	**13.94**	**5.3**	**11.37**	**9.51**	**13.5**	**10.33**	**29.10**			0.05
Octacosane	C_28_H_58_	394	0.65	1.76	1.21	1.43	0.97	0.26	2.32	0.91	1.53	1.32	1.67			0.05
Nonacosane	C_29_H_60_	408	4.69	8.04	5.07	6.65	7.03	2.91	6.8	5.6	7.14	6.22	5.03			0.07
Triacontane	C_30_H_62_	422	0.73	1.88	1.21	1.78	0.97	0.26	2.41	1.1	1.53	1.32	0.82			0.06
Hentriacontane	C_31_H_64_	436	5.67	7.16	5.67	7.48	7.03	3.8	7.88	6.36	6.83	7.02	2.23			0.08
Dotriacontane	C_32_H_66_	450	0.7	1.63	0.97	1.43	0.85	0.35	1.92	1.07	1.12	1.19	0.56			0.07
Tritriacontane	C_33_H_68_	464	6.81	6.78	6.52	6.89	8.49	5.03	8.04	7.49	6.91	8.87	3.31			0.08
Tetratriacontane	C_34_H_70_	478	0.42	1.38	0.48	0.95	0.36	0.18	1.13	0.56	0.49	0.53	0.34			0.06
Pentatriacontane	C_35_H_72_	492	1.01	2.39	1.21	1.43	2.43	0.26	1.54	0.6	2.21	1.99	1.03			0.06
Hexatriacontane	C_36_H_74_	506	0.3	0.88	0.97	0.83	0.49	0.97	0.67	0.31	0.48	0.4	0.16			0.06
Heptatriacontane	C_37_H_76_	520	0.18	0.75	0.24	0.48	0.12	N.D.	0.39	N.D.	0.22	0.26	N.D.			
Octatriacontane	C_38_H_78_	534	N.D.	0.38	N.D.	N.D.	0.12	N.D.	N.D.	N.D.	N.D.	0.13	N.D.			
Nonatriacontane	C_39_H_80_	548	N.D.	0.13	N.D.	N.D.	N.D.	N.D.	N.D.	N.D.	N.D.	N.D.	N.D.			
**Total**			**38.15**	**54.99**	**37.77**	**45.13**	**50.68**	**23.66**	**52.52**	**41.79**	**50.64**	**46.62**	**63.97**	**44.19**	**9.32**	**41.35**
CPI (o/e)^a^			7.5	4.4	4.6	4.1	8.5	5.5	3.6	5	6.1	5.9	5.1	5.5	1.5	
Plant Wax (%)^b^			28.97	33.33	24.68	27.61	39.22	17.44	29.21	28.88	34.96	32.64	42.88	**29.69**	**5.98**	0.01
Synthetic beewax (%)			9.18	21.66	13.09	17.52	11.46	6.22	23.31	12.91	15.68	13.97	21.08	**14.5**	**5.27**	41.34
**n** **-Alkenes**																
Tridecene	C_13_H_26_	182	N.D.	N.D.	N.D.	N.D.	N.D.	N.D.	N.D.	N.D.	N.D.	N.D.	N.D.			0.05
Tetradecene	C_14_H_28_	196	N.D.	N.D.	N.D.	N.D.	N.D.	N.D.	N.D.	N.D.	N.D.	N.D.	N.D.			0.06
Pentadecene	C_15_H_30_	210	N.D.	N.D.	N.D.	N.D.	N.D.	N.D.	N.D.	N.D.	N.D.	N.D.	N.D.			0.07
Hexadecene	C_16_H_32_	224	N.D.	N.D.	N.D.	N.D.	N.D.	N.D.	N.D.	N.D.	N.D.	N.D.	N.D.			0.06
Heptadecene	C_17_H_34_	238	N.D.	N.D.	N.D.	N.D.	N.D.	N.D.	N.D.	N.D.	N.D.	N.D.	N.D.			0.04
Octadecene	C_18_H_36_	252	N.D.	N.D.	N.D.	N.D.	N.D.	N.D.	N.D.	N.D.	N.D.	N.D.	N.D.			0.03
Nonadecene	C_19_H_38_	266	N.D.	N.D.	N.D.	N.D.	N.D.	N.D.	N.D.	N.D.	N.D.	N.D.	N.D.			0.03
Eicosene	C_20_H_40_	280	N.D.	N.D.	N.D.	N.D.	N.D.	N.D.	N.D.	N.D.	N.D.	N.D.	N.D.			0.01
Heneicosene	C_21_H_42_	294	N.D.	N.D.	N.D.	N.D.	N.D.	N.D.	N.D.	N.D.	N.D.	N.D.	N.D.			0.01
Docosene	C_22_H_44_	308	N.D.	N.D.	N.D.	N.D.	N.D.	N.D.	N.D.	N.D.	N.D.	N.D.	N.D.			0.01
Tricosene	C_23_H_46_	322	N.D.	N.D.	N.D.	N.D.	N.D.	N.D.	N.D.	N.D.	N.D.	N.D.	N.D.			N.D.
Tetracosene	C_24_H_48_	336	N.D.	N.D.	N.D.	N.D.	N.D.	N.D.	N.D.	N.D.	N.D.	N.D.	N.D.			N.D.
Pentacosene	C_25_H_50_	350	0.15	0.11	0.07	0.1	0.18	0.07	0.02	0.1	0.06	0.23	N.D.			N.D.
Hexacosene	C_26_H_52_	364	0.17	0.33	0.22	0.25	0.21	0.07	0.48	0.22	0.23	0.24	N.D.			N.D.
Heptacosene	C_27_H_54_	378	0.2	0.04	0.05	0.07	0.05	0.11	0.01	0.18	0.02	0.13	N.D.			N.D.
Octacosene	C_28_H_56_	392	0.25	0.03	0.04	0.05	0.01	0.04	0.04	0.25	0.01	0.07	N.D.			0.01
Nonacosene	C_29_H_58_	406	0.38	2.95	1.89	2.95	2.72	1.32	2.28	2.56	2.27	2.61	N.D.			N.D.
Triacontene	C_30_H_60_	420	0.36	0.75	0.54	0.72	0.41	0.1	0.61	0.38	0.62	0.56	0.19			N.D.
Hentriacontene	C_31_H_62_	434	9.2	6	6.32	8.27	6.37	5.07	8.33	2.46	5.82	7.26	0.95			N.D.
Dotriacontene	C_32_H_64_	448	0.84	1	0.84	1.05	0.81	0.44	1.27	0.95	0.84	1.17	0.37			N.D.
Tritriacontene	C_33_H_66_	462	**24.34**	**20.58**	**20.65**	**21.15**	**26.49**	**16.02**	**24.13**	**25.16**	**22.56**	**28.13**	12.93			N.D.
Tetratriacontene	C_34_H_68_	476	0.17	0.34	0.1	0.23	0.12	0.09	0.2	0.5	0.07	0.09	0.17			N.D.
Pentatriacontene	C_35_H_70_	490	0.51	0.84	0.63	0.62	0.9	0.42	3.5	0.78	6.23	0.69	0.13			N.D.
**Total**			**36.56**	**33.02**	**31.37**	**35.39**	**38.32**	**23.75**	**40.92**	**33.5**	**38.68**	**41.19**	14.75	**35.27**	**5.22**	**0.021**
CPI (o/e)^a^			19.5	12.4	17.1	14.5	23.4	31.4	14.8	13.6	20.7	19.5	19.08	18.6	5.7	0.6
**n** **-Alkanals**																
Octadecanal	C_18_H_36_O	268	0.34	0.13	0.25	0.35	0.08	0.26	0.06	0.05	0.1	0.18	N.D.			N.D.
Eicosanal	C_20_H_40_O	296	**0.65**	0.24	0.69	**0.9**	0.18	0.8	0.74	**0.15**	0.18	0.4	N.D.			N.D.
Docosanal	C_22_H_44_O	324	N.D.	0.01	0.01	0.02	N.D.	0.04	0.02	0.02	N.D.	0.01	N.D.			N.D.
Tetracosanal	C_24_H_48_O	352	0.02	**1.78**	1.39	0.43	**1.47**	0.86	**1.08**	0.13	**1.85**	**1.44**	T			N.D.
Hexacosanal	C_26_H_52_O	380	0.1	0.61	1	0.17	0.55	0.77	0.38	0.05	0.56	0.51	N.D.			N.D.
Octacosanal	C_28_H_56_O	408	0.11	0.31	0.94	0.14	0.35	0.96	0.21	0.03	0.37	0.29	N.D.			N.D.
Triacontanal	C_30_H_60_O	436	0.07	0.37	**1.61**	0.21	0.52	**1.87**	0.42	0.02	0.61	0.35	N.D.			N.D.
**Total**			**1.3**	**3.46**	**5.88**	**2.23**	**3.14**	**5.57**	**2.9**	**0.44**	**3.66**	**3.18**	T	**3.18**	**1.68**	**0**
**Methyl n-alkanoates**																
Methyl dodecannoate	C_13_H_26_O_2_	214	0.05	0.01	0.02	0.02	0.01	0.18	0.01	0.1	0.01	0.03	0.20			N.D.
Methyl tetradecanoate	C_15_H_30_O_2_	242	0.55	0.04	0.1	0.13	0.02	1.03	0.06	0.51	0.04	0.07	0.16			N.D.
Methyl hexadecanoate	C_17_H_34_O_2_	270	6.09	2.66	3.66	3.53	3.64	13.14	2.02	7.61	2.8	2.85	10.01			N.D.
Methyl octadecenoate	C_19_H_36_O_2_	296	6.65	0.5	1.38	1.91	0.5	13.42	0.67	4.04	0.3	0.78	0.74			N.D.
Methyl octadecanoate	C_19_H_38_O_2_	298	1.81	0.26	0.43	0.55	0.25	3.62	0.21	1.46	0.25	0.34	0.41			N.D.
Methyl eicosanoate	C_21_H_42_O_2_	326	0.46	0.05	0.1	0.13	0.04	0.92	0.05	0.28	0.02	0.07	0.01			N.D.
Methyl docosanoate	C_23_H_46_O_2_	354	0.14	0.04	0.04	0.05	0.04	0.35	0.02	0.09	0.02	0.05	0.10			N.D.
Methyl tetracosanoate	C_25_H_50_O_2_	382	0.2	0.11	0.07	0.08	0.11	0.46	0.06	0.15	0.11	0.21	0.43			N.D.
Methyl hexacosanoate	C_27_H_54_O_2_	410	0.05	0.03	0.01	0.01	0.01	0.09	0.01	0.03	0.01	0.04	0.08			N.D.
**Total**			**16.01**	**3.69**	**5.8**	**6.41**	**4.62**	**33.21**	**3.11**	**14.26**	**3.58**	**4.45**	**12.31**	**9.51**	**9.48**	
**Wax esters**																
Tetracosanyl hexadecanoate	C_40_H_80_O_2_	592	0.33	1.8	4.57	0.46	1.79	4.31	0.72	1.14	2.99	0.13	0.13			N.D.
Hexacosanyl hexadecanoate	C_42_H_84_O_2_	620	1.31	0.58	0.39	0.43	0.17	0.21	0.43	0.12	0.12	0.2	0.36			N.D.
Octacosanyl hexadecanoate	C_44_H_88_O_2_	648	0.58	0.2	0.08	0.19	0.08	0.04	0.26	N.D.	0.05	0.12	N.D.			N.D.
**Total**			**2.22**	**2.57**	**5.04**	**1.08**	**2.05**	**4.56**	**1.41**	**1.26**	**3.14**	**0.45**	**0.49**	**2.38**	**1.5**	
**Triterpenoids**																
β-Amyrone	C_30_H_48_O	424	0.21	0.18	0.22	0.12	0.11	0.08	0.05	N.D.	0.3	0.49	0.32			N.D.
α-Amyrone	C_30_H_48_O	424	0.26	0.63	0.48	0.12	0.61	0.09	0.11	N.D.	0.64	1.59	2.33			N.D.
β-Amyrin	C_30_H_50_O	426	0.16	0.11	0.22	0.05	0.1	0.02	0.12	N.D.	0.12	0.46	0.15			N.D.
α-Amyrin	C_30_H_50_O	426	0.16	0.09	0.13	0.01	0.23	0.03	0.07	N.D.	0.11	0.53	1.22			N.D.
β-Amyryl acetate	C_32_H_52_O_2_	468	0.11	0.36	0.21	0.05	1.1	0.02	0.07	N.D.	0.21	1.24	0.65			N.D.
**Total**			**0.9**	**1.37**	**1.25**	**0.34**	**2.15**	**0.24**	**0.43**		**1.38**	**4.31**	**4.76**	**1.38**	**1.27**	
**Hopane Biomarkers**																
Trisnorneohopane	C_27_H_46_	370	0.09	N.D.	0.27	0.1	N.D.	0.24	N.D.	0.13	N.D.	0.03	N.D.			0.02
17α(H)-Trisnorhopane	C_27_H_46_	370	0.18	N.D.	0.35	0.25	N.D.	0.41	N.D.	0.29	N.D.	0.03	N.D.			0.03
17α(H),21β(H)-Norhopane	C_29_H_50_	398	0.95	N.D.	1.76	1.18	N.D.	2.42	N.D.	1.32	N.D.	0.16	N.D.			0.18
17α(H),21β(H)-Hopane	C_30_H_52_	412	0.8	N.D.	1.85	0.95	N.D.	1.98	N.D.	1.17	N.D.	0.13	N.D.			0.18
17α(H),21β(H)-22S-Homohopane	C_31_H_54_	426	0.49	N.D.	0.99	0.55	N.D.	1.18	N.D.	0.73	N.D.	0.07	N.D.			0.13
17α(H),21β(H)-22R-Homohopane	C_31_H_54_	426	0.32	N.D.	0.64	0.39	N.D.	0.81	N.D.	0.48	N.D.	0.04	N.D.			0.08
Gammacerane	C_30_H_52_	412	0.02	N.D.	0.01	0.02	N.D.	0.11	N.D.	0.07	N.D.	0.01	N.D.			0.01
17α(H),21β(H)-22S-Bishomohopane	C_32_H_56_	440	0.3	N.D.	0.59	0.34	N.D.	0.74	N.D.	0.47	N.D.	0.04	N.D.			0.13
17α(H),21β(H)-22R-Bishomohopane	C_32_H_56_	440	0.35	N.D.	0.83	0.55	N.D.	0.71	N.D.	0.56	N.D.	0.05	N.D.			0.08
17α(H),21β(H)-22S-Trishomohopane	C_33_H_58_	454	0.31	N.D.	0.58	0.43	N.D.	0.75	N.D.	0.51	N.D.	0.04	N.D.			0.09
17α(H),21β(H)-22R-Trishomohopane	C_33_H_58_	454	0.22	N.D.	0.54	0.26	N.D.	0.5	N.D.	0.31	N.D.	0.04	N.D.			0.06
17α(H),21β(H)-22S-Tetrakishomohopane	C_34_H_60_	468	0.27	N.D.	0.45	0.33	N.D.	0.7	N.D.	0.44	N.D.	0.05	N.D.			0.06
17α(H),21β(H)-22R-Tetrakishomohopane	C_34_H_60_	468	0.2	N.D.	0.72	0.42	N.D.	0.66	N.D.	0.28	N.D.	0.09	N.D.			0.05
17α(H),21β(H)-22S-Pentakishomohopane	C_35_H_62_	482	0.35	N.D.	0.76	0.43	N.D.	0.79	N.D.	0.41	N.D.	0.05	N.D.			0.07
17α(H),21β(H)-22R-Pentakishomohopane	C_35_H_62_	482	0.18	N.D.	0.52	0.27	N.D.	0.49	N.D.	0.19	N.D.	0.03	N.D.			0.05
**Total**			**5.02**		**10.87**	**6.46**		**12.48**		**7.36**		**0.87**		**6.15**	**4.67**	**1.23**
C_31_ S/(R+S)			0.61		0.61	0.58		0.59		0.6		0.61		0.6	0.01	0.61
C_32_ S/(R+S)			0.46		0.41	0.39		0.51		0.46		0.44		0.45	0.05	0.61

N.D. = not detected.

^a^ = $CPI_{\left(o/e\right)}=\left(\frac{\sum nC_{odd}}{\sum nC_{even}}\right)$

^b^ = Calculated as wax C_n_ = [C_n_]-[(C_n+1_)+(C_n-1_)/2], [24]. Bold type = compound totals and maximum concentration for each compound group.

### n-Alkanes

Hydrocarbon alkanes are major fraction in propolis [14–17]. The n-alkanes are derived from both biogenic and anthropogenic sources and can be differentiated based on their distribution patterns in samples. Thus, they are useful to assess their source in different samples. Key parameters associated with n-alkane sources and characteristics are the well-established carbon preference index (CPI), and the carbon number maximum (C_max_) of the most abundant n-alkane in the homologous series [18]. The CPI of n-alkanes can be used to assess the influence of biogenic and anthropogenic inputs [19]. The relative n-alkane concentrations ranged from 23.7% to 55.0% with a mean of 44.2+9.3% (Table 1) for propolis. These values were less than the control sample (64.0%). The odd-numbered n-alkanes were dominant over the entire range and the CPI_o/e_ varied from 3.6 to 7.7 (mean = 5.5+1.5, Table 1), which is similar to the value of the control (5.1). They have C_max_ at 27 and range from C_17_ to C_40_ (Table 1 and Fig 2). This indicates that vascular plants are major sources of the n-alkanes, with some contribution from synthetic bee wax for these propolis samples. Plant wax n-alkanes have a C_max_ in the range of 25–31, which is dependent on the plant species as well as the season and locality [20–22].

For the asphalt, the n-alkanes range from C_12_ to C_36_ with C_max_ at 15 (Fig 3). Their relative concentration was 41.3% and the CPI_o/e_ ~1.0. The asphalt also exhibits an unresolved complex mixture (UCM) of branched and cyclic hydrocarbons with a bimodal distribution and maxima at alkane retention index of C_16_ (major) and C_32_ (minor, Fig 3A). This UCM, oily in terms of fluidity, is not significant in the propolis sample extracts.

**Fig 4:**
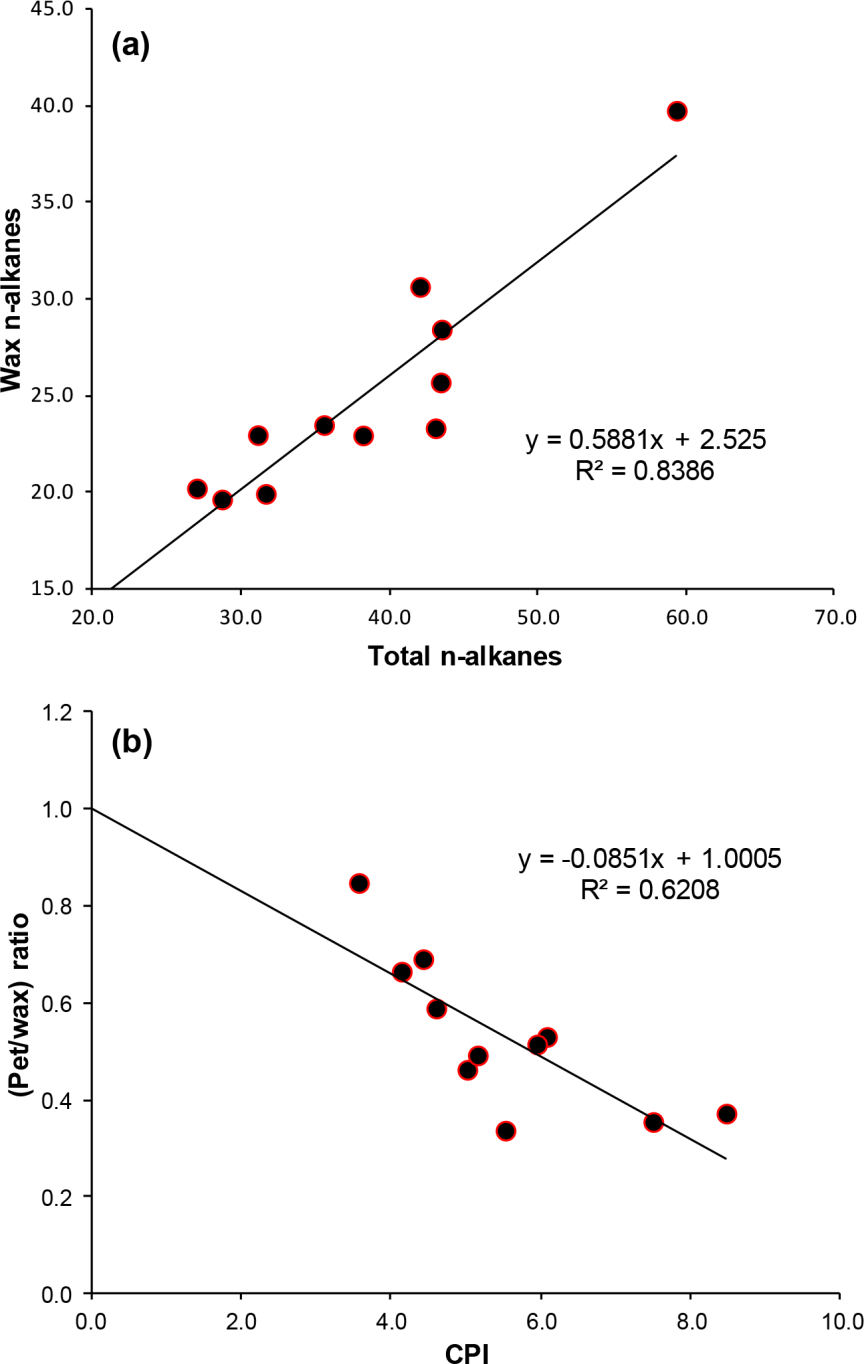
Plots showing the relationships between (a) wax n-alkanes versus total n-alkanes and (b) petroleum-to-biogenic n-alkane ratios versus CPI of n-alkanes.

In order to better evaluate the relative input from various sources, the concentrations of plant wax versus petroleum derived n-alkanes [23], and their percentage of the total n-alkanes were calculated (Table 1). The amounts of plant wax n-alkanes range from 17.8% to 33.6% of the total extracts, whereas the petroleum dervied inputs ranged from 6.2% to 21.7%. There is a significant correlation (R^2^ = 0.84) between wax n-alkanes (Fig 4A) and the total n-alkanes, which suggests that terrestrial vegetation wax is the major contributor to the total n-alkanes in the propolis samples. The CPI also shows a negative correlation (R^2^ = 0.62) with the ratios of petroleum-to-plant wax (Fig 4B), which supports the effectiveness of CPI as an indicator of biogenic sources from plant waxes versus petroleum hydrocarbon inputs.

### n-Alkenes

Recent studies showed that n-alkenes are present in both bee wax and propolis [16,17,24]. They are mainly hentriacontene (C_31:1_) and tritriacontene (C_33:1_) [24]. The n-alkenes (Δ^1^ or Δ^9^) were major compounds in these propolis samples and ranged from C_25_ to C_35_ with a C_max_ at 33. The relative concentrations varied from 23.8% to 41.2% with a mean of 35.3+5.2%, and were higher than their relative concentration (14.8%) in the control sample. The odd carbon numbered n-alkenes were dominant with a CPI_o/e_ of 12.4 to 31.4 (mean = 18.6+5.7), and similar to the value (19.1) of the control. The distribution of n-alkenes with major concentrations of the odd numbered homologues and C_max_ at 33 suggests an origin from insect wax [25,26], possibly derived by alteration of long chain n-alkanols. The asphalt extract contained only traces on n-alkenes mainly from C_13_-C_23_ (Table 1).

### n-Alkanals

n-Alkanals were detected in these propolis samples as minor compounds. They ranged from 0.44% to 5.9% with a mean value of 3.2+1.7% of the total extracts (Table 1) and had various chain length dominances. They were trace components in the control sample. Only even-numbered n-alkanals were observed ranging from C_24_ to C_30_. The n-alkanals are intermediates in the biochemical synthesis of n-alkanols and are minor components of higher plant waxes [27]. The n-alkanals were not detectable in the asphalt samples.

### Methyl n-alkanoates

The methyl n-alkanoates were also significant compounds at relative concentrations of 3.1% to 33.2% with a mean of 9.5+9.5% (Table 1), and in the control sample with a relative concentration of 12.3%. They ranged from C_13_ to C_27_ with C_max_ at methyl n-hexadecanoate (16) and n-octadecenoate (18:1). The methyl n-alkanoates had solely an even carbon number predominance (as the alkanoic acids, Table 1), indicating that they are originally from natural biogenic sources [28,29]. Alkanoic acids, with hexadecanoic as major acid, have been reported in other propolis samples [16,17]. No methyl n-alkanoates nor n-alkanoic acids were detectable in the asphalt extract.

### Long chain wax esters

Long chain wax esters were detected in these samples with relative concentrations of 0.45% to 5.04%, mean 2.4+1.5%, and consisting mainly of tetracosyl-, hexacosyl- and octacosyl hexadecanoates, where hexacosyl hexadecanoate was dominant (Table 1). They were relatively low (0.49%) in the control sample from Al-Bahah, and have also been reported as minor components of other propolis samples [17]. Long chain wax esters are likely derived from waxes secreted by the bees [30] or from lipid components of vascular plants of the region [31–33]. Waxes secreted by bees contain more than 15% of wax esters and generally include tetradecyl dodecanoate, tetradecanoate and hexadecanoate, as well as hexadecyl tetradecanoate and hexadecanoate [34]. The non-hydrocarbon components of higher plant waxes are generally alcohols (40%) in younger plants and mainly wax esters (42%) in older plants [35,36]. Besides the geographical location, the vegetation wax ester composition also depends on the plant species [5]. Wax esters were not detectable in the asphalt extract.

### Triterpenoids

Triterpenoids were detected in the propolis samples with relative concentrations of 0.24% to 4.3%, mean 1.4+1.3%, consisting mainly of α- and β-amyrones, amyrins, and amyryl acetates (Table 1). They were significant with a relative concentration of 4.67% in the control sample. α-Amyrone and β-amyryl acetate were the major triterpenoids (Table 1). The variation in the contents is likely due to different plant species of the same family. These triterpenoids were also reported for propolis samples from Brazil, Egypt, Cuba and Ethiopia [37–41], but were not detectable in the asphalt. Obviously, the main source of triterpenoids in propolis is from the regional vegetation.

### Hopanes

Hopanes were found in many of these propolis samples with concentrations ranging from 0.0 to 12.5% (mean = 6.2+4.7%, Table 1). These compounds were not detected in the control sample from the Al-Bahah region. The presence of hopane hydrocarbons in propolis has not been reported and is of interest because it indicates an input of petroleum products, especially less volatile sticky material such as tar and asphalt. Fossil or geo-hopanes are usually resistant to degradation and alteration in the environment and can be used as tracers to indicate pollution from the utilization of petroleum and its products in environmental samples [42–44]. The hopane hydrocarbons in these samples had the thermodynamically more stable 17α(H),21β(H) configuration, ranging from C_27_ to C_35_ (no C_28_), with C_30_ maximum, and minor 17β(H),21α(H)-hopanes (Figs 2C vs. 3C), typical of crude oils and products [42–45]. These cyclic hydrocarbons are derived from the diagenetic interconversion of the 17β(H),21β(H)-hopane precursors of bacterial origins over geological times [45]. The distributions of the hopanes > C_31_ all showed the typically mature C-22 R/S epimer pairs [43,46]. The ratios of C-22 S/(S+R) for the C_31_ and C_32_ hopanes ranged from 0.59 to 0.60 (mean = 0.60+0.01) and from 0.39 to 0.46 (mean 0.45+0.05) (Table 1), respectively. These values are in the same range as hopanes in mature crude oils or hydrothermal petroleums [45–49], supporting their origin from petroleum products. The asphalt sample from the hive region had a relative concentration of hopanes of 1.23% (Fig 3C). The C-22 S/(S+R) ratio for C_31_ and C_32_ was 0.61 for both, similar as the distribution and ratio of the propolis samples. This confirms that the bees have used asphalt as one of the ingredients to make propolis for their hives, particularly with the other natural sources being scarce.

Steranes, the other fossil fuel tracers originating from sterols over geological times, are also common in asphalts [44,45]. However, steranes were not detectable in this asphalt sample nor in any of the propolis samples of this study, providing indirect evidence, in conjunction with the presence of hopanes and petroleum derived n-alkanes, that asphalt was utilized as a propolis component.

Soil and sand particles contain a variety of anthropogenic and natural organic components, and in urban areas can be considered pollutant collectors [50–52]. Small particles of soil and sand can be resuspended into the air and transported by wind to different places. To confirm that dust from the surrounding area or long distance transported atmospheric particulate matter (PM) are not the contributors of hopanes in these propolis samples, surface soil samples were taken from two areas in Riyadh as well as an atmospheric PM sample (S1 Fig). The major anthropogenic compounds of the soil and atmospheric PM samples were plasticizers for the two surface soil and the atmospheric PM samples, ranging from 29.3% to 74.7% (S2 Fig and S1 Table). n-Alkanes were 11% and 17% in the D-S and O-S soil samples, respectively, and 26% in the atmospheric PM. The relative concentrations of the unresolved complex mixture (UCM) were relatively lower in the soil samples (2.2% for D-S and 5.09% for O-S) than in the atmospheric PM (29.3%). The hopane and sterane biomarkers were detected at trace amounts in the soil sample near the bee hives and as major compounds in the soil sample from the city center and in the atmospheric PM (S3 Fig and S1 Table). The absence of plasticizers and sterane biomarkers, which were major compounds in the soil (O-S) and atmospheric PM samples, in propolis and asphalt verify that the source of the hopanes in propolis is mainly from asphalt collected by bees and not from transported dust. Furthermore, if the major source of contaminants in propolis were from dust then hopanes should have been detected in all propolis samples. Further support that dust is not a major contributor to propolis contaminants is the presence of traces of both hopane and sterane biomarkers in the surface soil sample from the site near the bee hives, which is not consistent with the occurrence of hopanes as major compounds and the absence of steranes in the propolis. Also, it is notable that traces of UCM were detected only in propolis samples that contain hopanes (S1 Table), indicating that both are originally from the asphalt.

### Statistical analysis

**Fig 5:**
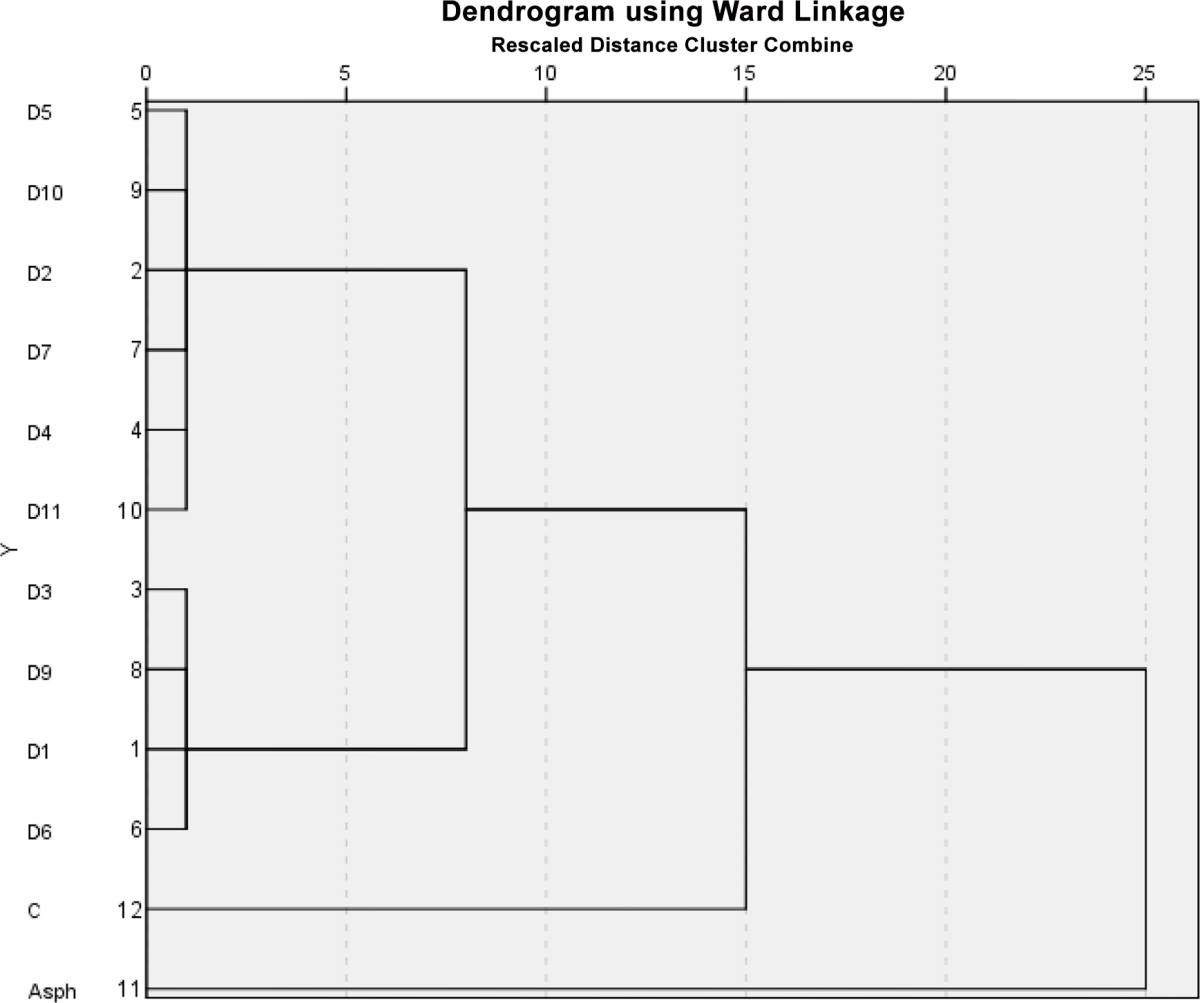
Plot showing the statistical output of cluster analysis.

**Fig 6:**
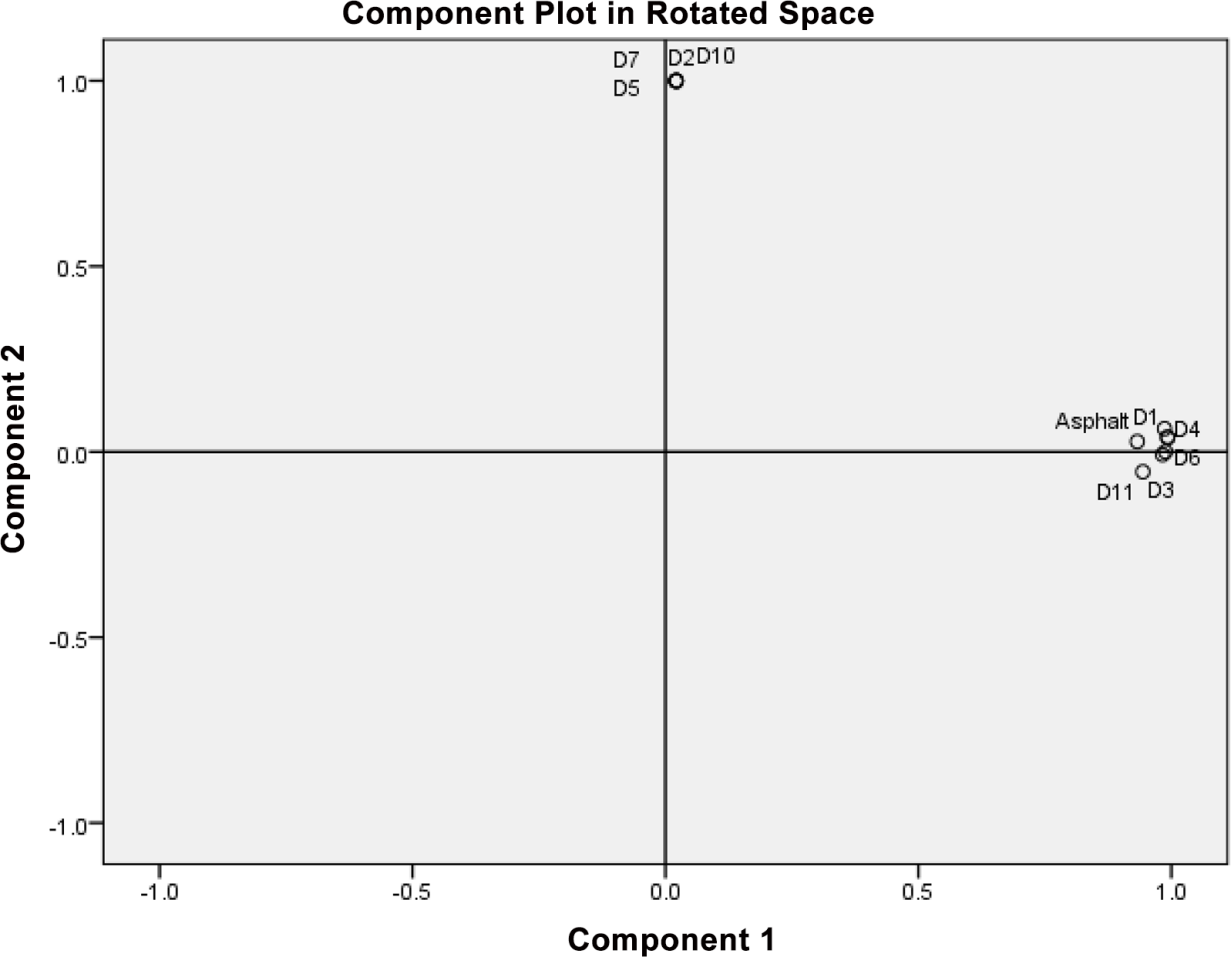
Plot showing the statistical output of principal component analysis (PCA).

The output of the cluster analysis is shown in Fig 5 where four separate sample clusters were recognized. The first cluster included six propolis samples, the second one included four propolis samples, and third and fourth included the asphalt and the control as single samples. The underlying structure of the data set was explained by the output of the PCA in Fig 6. The ordination plots of the propolis samples showed a clear separation of the samples: asphalt, D1, D3, D4, D6, D9 and D11 from the samples: control, D2, D5, D7 and D10. The two principal vectors (Fig 6) showed only two clusters along axes 1 and 2. The separation in the data set was evident and confirmed a dissimilarity among the different propolis samples, which was clearly shown in Fig 6. The reproduced correlation coefficients between asphalt and propolis D1, D3, D4, D6, D9 and D11 were significant (R^2^ = 0.917–0.927). The Quartimax with Kaiser Normalization of components 1 and 2 ranged from 0.986 to 0.993 and from-.0.054 to 0.037 for the samples D1, D3, D4, D9 and D11, respectively.

**Fig 7:**
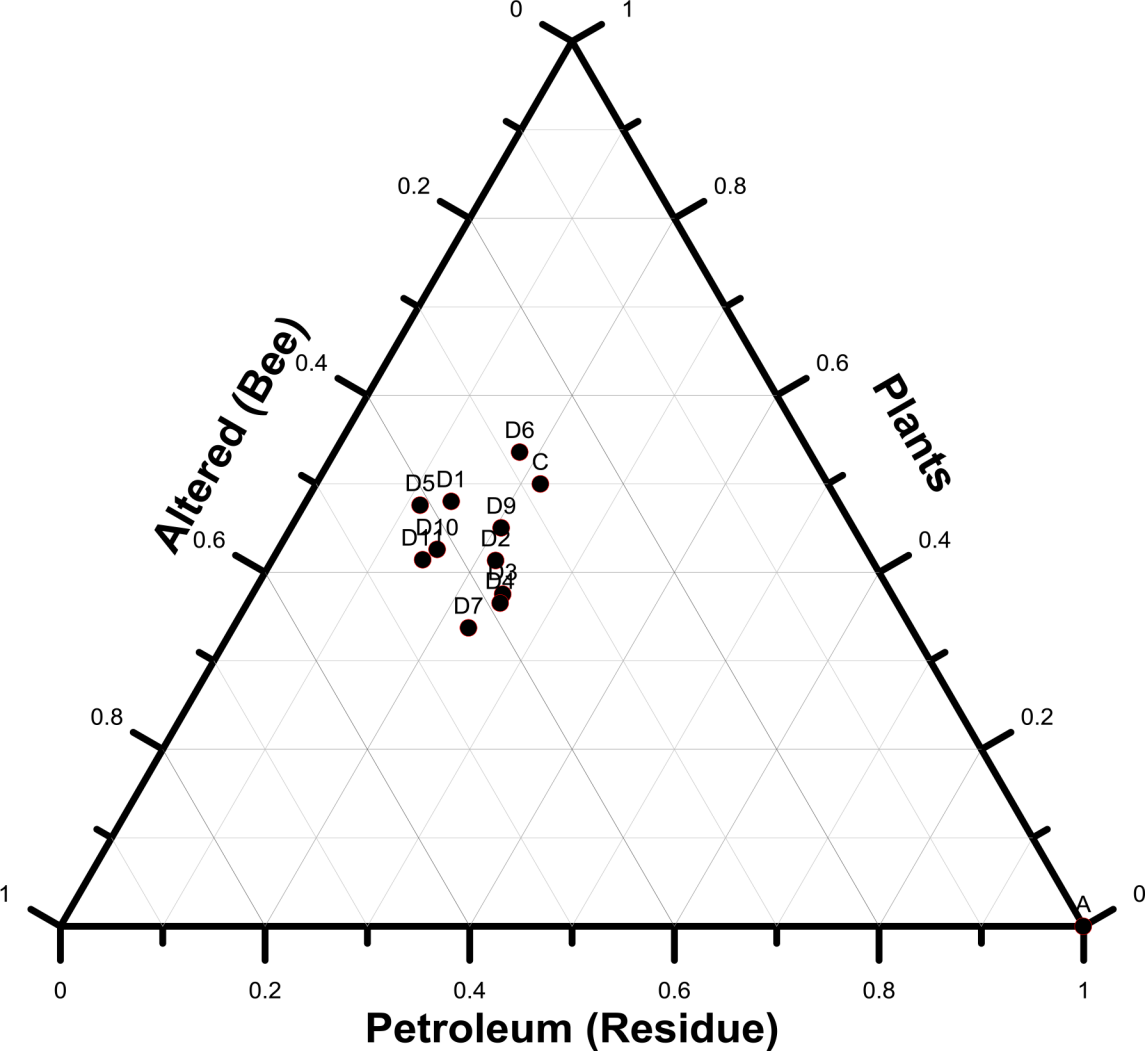
Ternary diagram showing the compound compositions from petroleum residues, altered products and natural plant wax.

Obviously, the sources of organic matter of these propolis samples are mainly compounds from terrestrial plants, some altered by bees or chemical processes, and asphalt. The inputs from plant wax were calculated as the sum of plant wax n-alkanes, triterpenoids and wax esters; the compounds altered by bees as the sum of n-alkenes, n-alkanals and methyl n-alkanoates; and the inputs from asphalt as the sum of hopanes, UCM and petroleum n-alkanes. The contribution of asphalt was evident in the propolis samples as shown in Fig 7 and ranged from 11.5% to 24.0% (mean = 18.8+4.5%). The local vegetation contributed from 34.2% to 48.1% (mean = 42.8+6.6%) and the compounds altered by chemical processes and/or bee metabolism ranged from 29.3% to 44.4% (mean = 38.4+4.7%) (Fig 7). The collection of asphalt by bees to make propolis, when resin is scarce, is likely driven by the similar aroma and/or dark color of resin and asphalt or the stick factor (viscosity).

## Conclusion

The hexane-extractable aliphatic lipids present in propolis samples from Riyadh and an asphalt sample from the hive vicinity have been characterized using GC–MS techniques. Inputs of wax from vascular higher plants and asphalt residues, as well as compounds altered by bee metabolism are obvious in the propolis samples. The contributions from asphalt are detectable as confirmed by the presence of hopanes and petroleum-derived n-alkanes. The n-alkanes (odd carbon dominance >C_25_), wax esters and triterpenoids indicate a dominant input from vascular higher plant wax, whereas n-alkenes, methyl n-alkanoates and n-alkanals likely maybe compounds altered by the bees.

## Supporting Information

S1 Fig Map showing the site locations of the surface soil samples D-S and O-S and the atmospheric particulate matter (D-PM) sample. (DOC)

S2 Fig GC-MS total ion current traces of total SOM extracts of soil samples from Riyadh: (a) D-S from the BRU site near the honey bee hives and (b) from the city center (D-S), and atmospheric PM from the two-story building near BRU (c) D-PM (Numbers refer to the carbon chain length of n-alkanes and symbols are I = isobutyl-, II = dibutyl-, III = diethyhexyl phthalate, A = Triphenyl phosphate, B = Monotolyl diphenyl phosphate, C = Monophenyl ditotyl phosphate, H = hopane). (DOC)

S3 Fig Examples of typical GC-MS key ion plots for hopanes, *m/z* 191: (a) D-S, (b) O-S and (c) D-PM, and for steranes, *m/z* 217 and 218: (e) D-S, (f) O-S and (g) D-PM. (DOC)

S1 Table Relative concentrations (%) of major anthropogenic components in propolis, soil, and air particulate matter (PM) samples from Riyadh, Saudi Arabia. (DOC)
