# Supplementary material for: Organic Tracers from Asphalt in Propolis Produced by Urban Honey Bees, Apis mellifera Linn
Source: PLoS One. 2015 Jun 15;10(6):e0128311. doi: 10.1371/journal.pone.0128311 (PMC4468070; doi:10.1371/journal.pone.0128311)
Supplement: S3 Fig — (DOC) [file pone.0128311.s003.doc]

S3 Fig.
