# Supplementary material for: Organic Tracers from Asphalt in Propolis Produced by Urban Honey Bees, Apis mellifera Linn
Source: PLoS One. 2015 Jun 15;10(6):e0128311. doi: 10.1371/journal.pone.0128311 (PMC4468070; doi:10.1371/journal.pone.0128311)
Supplement: S1 Table — (DOC) [file pone.0128311.s004.doc]

S1 Table. Relative concentrations (%) of major anthropogenic components in propolis, soil, and air particulate matter (PM) samples from Riyadh, Saudi Arabia.

| **Compounds** | **D1** | **D2** | **D3** | **D4** | **D5** | **D6** | **D7** | **D9** | **D10** | **D11** | **C** | **Asphalt** | **D-S** | **O-S** | **D-PM** |
| --- | --- | --- | --- | --- | --- | --- | --- | --- | --- | --- | --- | --- | --- | --- | --- |
|  |  |  |  |  |  |  |  |  |  |  |  |  |  |  |  |
| **n-Alkanes** |  |  |  |  |  |  |  |  |  |  |  |  |  |  |  |
| Total | 38 | 55 | 38 | 45 | 51 | 24 | 53 | 42 | 51 | 47 | 64 | 41 | 11 | 17 | 26 |
| Range | 17-37 | 20-40 | 20-37 | 18-37 | 21-38 | 18-36 | 18-37 | 17-36 | 17-37 | 21-38 | 20--36 | 12-36 | 21-33 | 14-37 | 19-33 |
| C_max_ | 27 | 27 | 27 | 27 | 27 | 27 | 27 | 27 | 27 | 27 | 27 | 15 | 29 | 29 | 25 |
| CPI (o/e) | 7.5 | 4.4 | 4.6 | 4.1 | 8.5 | 5.5 | 3.6 | 5 | 6.1 | 5.9 | 5.1 | 1.0 | 3.5 | 1.3 | 1.2 |
|  |  |  |  |  |  |  |  |  |  |  |  |  |  |  |  |
|  |  |  |  |  |  |  |  |  |  |  |  |  |  |  |  |
| **Hopane biomarkers** |  |  |  |  |  |  |  |  |  |  |  |  |  |  |  |
| Total | 5.02 | N.D. | 10.9 | 6.46 | N.D. | 12.5 | N.D. | 7.36 | N.D. | 0.87 | N.D. | 1.23 | T | 6.94 | 9.1 |
| Range | 27-35 |  | 27-35 | 27-35 |  | 27-35 |  | 27-35 |  | 27-35 |  | 27-35 |  | 27-35 | 27-35 |
| C_max_ | 29,30 |  | 29,30 | 29,30 |  | 29,30 |  | 29,30 |  | 29,30 |  | 29,30 |  | 29 | 29 |
| C_31_ S/(R+S) | 0.61 |  | 0.61 | 0.58 |  | 0.59 |  | 0.6 |  | 0.61 |  | 0.61 |  | 0.55 | 0.56 |
| C_32_ S/(R+S) | 0.46 |  | 0.41 | 0.39 |  | 0.51 |  | 0.46 |  | 0.44 |  | 0.61 |  | 0.63 | 0.67 |
|  |  |  |  |  |  |  |  |  |  |  |  |  |  |  |  |
| **Sterane biomarkers** |  |  |  |  |  |  |  |  |  |  |  |  |  |  |  |
| Total | N.D. | N.D. | N.D. | N.D. | N.D. | N.D. | N.D. | N.D. | N.D. | N.D. | N.D. | N.D. | T | 2.06 | 3.72 |
| Range |  |  |  |  |  |  |  |  |  |  |  |  |  | 27-29 | 27-29 |
| C_max_ |  |  |  |  |  |  |  |  |  |  |  |  |  | 27 | 29 |
|  |  |  |  |  |  |  |  |  |  |  |  |  |  |  |  |
| **Plasticizers** |  |  |  |  |  |  |  |  |  |  |  |  |  |  |  |
| Total | N.D. | N.D. | 0.47 | N.D. | N.D. | N.D. | N.D. | 0.15 | N.D. | N.D. | N.D. | N.D. | 53.5 | 74.7 | 29.3 |
|  |  |  |  |  |  |  |  |  |  |  |  |  |  |  |  |
| UCM | 1.8 | N.D. | 3.0 | 2.7 | N.D. | 3.4 | N.D. | 3.1 | N.D. | 1.0 | N.D. | 57.5 | 2.2 | 5.09 | 24.8 |
| U:R | 0.02 |  | 0.03 | 0.03 |  | 0.03 |  | 0.03 |  | 0.01 |  | 1.35 | 0.20 | 0.56 | 0.33 |

N.D. = not detected

T = trace

C_max_, CPI, UCM and U:R are as defined in the text and Table 1
